# Epidemic Small-Pox

**Published:** 1846-02

**Authors:** 


### EPIDEMIC SMALL-POX.

Small-pox has prevailed to a very unusual extent in this county for several months past. In every quarter of it cases have arisen, and excited much alarm, although, for the most part, they have assumed a mild form, the subjects having previously had the disease, or been vaccinated. People are strangely negligent of vaccination, and alarms of small-pox are necessary, now and then, to awaken them to a sense of its importance. The disease is attended with very little danger or suffering, in persons who have been impressed with the vaccine virus. Such cases scarcely require medical treatment, and are followed by no disfiguration. The profession ought to take advantage of the present excitement on the subject, to diffuse the prophylactic as universally as possible.

